# Accessibility and readability of online patient education on cutaneous lymphomas

**DOI:** 10.1016/j.jdin.2023.07.010

**Published:** 2023-07-28

**Authors:** Celine M. Schreidah, Lauren M. Fahmy, Brigit A. Lapolla, Emily R. Gordon, Bradley D. Kwinta, Larisa J. Geskin

**Affiliations:** a Columbia University Vagelos College of Physicians and Surgeons, New York, New York; b Department of Dermatology, Columbia University Irving Medical Center, New York, New York

**Keywords:** accessibility, cutaneous B-cell lymphoma, cutaneous lymphoma, cutaneous T-cell lymphoma, digital health, patient education, readability

## Abstract

**Background:**

Patients facing a cutaneous lymphoma diagnosis frequently turn to the internet for information but finding patient-accessible education may be a challenge.

**Objective:**

To investigate accessibility and readability of patient-oriented online education on cutaneous lymphomas, including cutaneous T-cell and B-cell lymphoma subtypes.

**Methods:**

This study queried a search engine for 11 cutaneous lymphoma terms, resulting in 1083 webpages. Webpages were screened using defined inclusion/exclusion criteria; literature directed to physicians and scientists was excluded. Webpages were stratified by academic/nonacademic and dermatology/nondermatology hosts and assessed by order of appearance. Readability, including text complexity, was analyzed for grade level understanding using 5 established calculators. Overall readability was assessed by Flesch–Kincaid Reading Ease.

**Results:**

Academic webpages had earlier order of appearance. There was a dearth in dermatology-hosted webpages. Rarer cutaneous lymphomas yielded fewer patient-accessible results. Search term readability significantly exceeded the American Medical Association–recommended sixth grade level (*P* < .001∗), with higher grade levels for cutaneous T-cell lymphoma subtype webpages than cutaneous B-cell lymphoma subtypes.

**Limitations:**

Webpage quality, accuracy, and language were not assessed.

**Conclusion:**

Current online education for cutaneous lymphomas exceeds the American Medical Association’s maximum readability recommendation. There is a need for more patient-accessible education amidst predominance of scientific literature, greater dermatology host websites, and enhanced readability of existing online education.

**Capsule Summary**

• Patients frequently access the internet for education regarding cutaneous lymphoma diagnosis and treatments; however, scientific literature dominates searches, and patient-oriented education readability has not been evaluated.
• There is scarcity of readable, patient-oriented online education on cutaneous lymphomas, particularly rarer subtypes. Readability of patient-accessible online education universally exceeded American Medical Association’s maximum recommendations.

## Introduction

Most patients in the United States view online health resources upon diagnosis and for making healthcare decisions. Health Information National Trends Survey administered several times from 2008 to 2017, showed that nearly 69% of patients first use the internet to search for health information.^1^ This trend is particularly important for patients with rare diseases, such as cutaneous lymphomas. Cutaneous lymphomas are malignancies of T- and B-lymphocytes which primarily impact the skin. Primary cutaneous B-cell lymphomas (CBCLs) comprise approximately 25% of all primary cutaneous lymphomas in the United States, with cutaneous T-cell lymphomas (CTCLs) comprising nearly all the rest.^2^ Although patients with early stage CTCL and CBCL generally have a good prognosis and an indolent disease course,^3^ many patients experience significant health distress.^4^ This may, in part, be because of the uncertainty that accompanies a cancer diagnosis which may prompt patients to seek out additional information to make sense of their diagnosis and treatment options.

Online patient educational materials can be hosted or created by academic sources, such as hospitals, institutions, physicians, and academic organizations, or by nonacademic sources, primarily news sites and industry. Nonacademic pages may also provide insight into the patient’s experience through personal stories on blogs and health sites. Availability of online materials hosted on dermatologic websites is of particular interest, given most diagnostic decision-making and multidisciplinary care coordination for cutaneous lymphomas are directed by dermatologists.

It is imperative that patient-oriented online education on cutaneous lymphomas is written in an accessible fashion, particularly given the complexity and rarity of many cutaneous lymphoma subtypes. The American Medical Association (AMA) recommends health information be presented at a maximum sixth grade reading level.^5^ The AMA recommendation for clinical practices with a high percentage of patients with limited literacy is third to fifth grade, noting the average reading skill of Medicaid enrollees is fifth grade level.

This study sought to assess both accessibility and readability of online educational materials that patients leverage in their pursuit of knowledge on cutaneous lymphomas, particularly CTCL and CBCL subtypes. To our knowledge, such a comprehensive and comparative investigation has not yet been conducted and published in the literature on cutaneous lymphomas.

## Methods

Internet searches for terms related to CTCL, CBCL, and their most common respective subtypes were performed through Google on incognito mode (limiting personalization/bias). Search terms were under 3 main categories: “cutaneous lymphoma” broadly, CTCL, and CBCL. For CTCL, cutaneous T-cell lymphoma, mycosis fungoides, Sézary syndrome, folliculotropic mycosis fungoides, lymphomatoid papulosis, and anaplastic large-cell lymphoma were queried.^6^ For CBCL, cutaneous B-cell lymphoma, follicle center lymphoma, marginal zone lymphoma, and diffuse large B-cell lymphoma were queried.^2^

The first 10 pages of search results, as available, for each of the 11 total search terms were screened by inclusion and exclusion criteria. Criteria for exclusion included academic journal articles, articles with a paywall or subscription for access, videos (nonstill web media), books and encyclopedias, advertised search results, repeated links among search results, non-English articles, and clinic advertisements not featuring education. These criteria were set to exclude search results deemed inaccessible to patients, redundant, or not containing information on the term of interest.

Each included webpage was analyzed for academic/nonacademic hosting, order of appearance among search results, and dermatology/nondermatology hosting. Readability was assessed by established grade level calculators (Flesch–Kincaid Grade Level, Gunning Fog Score, SMOG Index, Coleman Liau Index, and Automated Readability Index) along with the Flesch–Kincaid Reading Ease score. Flesch–Kincaid Grade Level calculates readability as a grade-equivalent level, reporting American school grade required for reader comprehension.^5^^,^^7^ The Gunning Fog Index estimates years of formal education needed to comprehend a text passage upon initial reading, with a scale from 0 to 20.^7^^,^^8^ It scores text with short sentences in plain English as better than longer sentences in complex language; the ideal Gunning Fog Index grading score is said to be 7 or 8, with anything higher than 12 deemed too complex for most people to read.^7^ The SMOG Index estimates years of education the average person needs to comprehend a piece of writing and was founded to improve upon the Gunning Fog Index.^7^ The Coleman Liau Index is designed to evaluate the American grade level necessary to understand a text, employing a formula based on characters (word length) instead of other syllable-based readability indicators.^7^ The Automated Readability Index is a grade level calculator derived from ratios representing sentence difficulty (number of words per sentence) and word difficulty (number of letters per word).^7^ Readability and text complexity were assessed via the WebFX Readability Test.^7^ Descriptive and comparative statistics were conducted in Microsoft Excel Version 16.66.1.

## Results

### Search results, order of appearance, and website hosting

Following screening of 1083 webpages for the 11-term web searches, 346 total webpages were included. There were 259 academic and 87 nonacademic webpages identified among the 11 cutaneous lymphoma search terms. Summary and comparative *t* test statistics for academic vs nonacademic webpages were computed for order of appearance (Table I).

**Table I:** Search page order of appearance among academic and nonacademic pages

“Search term” or Group	Academic average (minimum, maximum)	Nonacademic average (minimum, maximum)	*P* value
“Cutaneous Lymphoma”	4.50 (1, 10)	7.50 (4, 10)	.003∗
“Cutaneous T-Cell Lymphoma”	4.19 (1, 10)	6.13 (2, 10)	.029†
“Mycosis Fungoides”	4.60 (1, 10)	4.29 (1, 10)	.937
“Sezary Syndrome”	4.90 (1, 10)	4.73 (1, 10)	.997
“Folliculotropic Mycosis Fungoides”	1.75 (1, 3)	7.29 (4, 9)	<.001∗
“Lymphomatoid Papulosis”	2.67 (1, 9)	5.20 (3, 7)	.097
“Anaplastic Large-Cell Lymphoma”	3.60 (1, 9)	5.00 (1, 10)	.181
“CTCL” and *Subtypes*	3.62 (1.00, 8.50)	5.73 (2.29, 9.43)	.010†
“Cutaneous B-Cell Lymphoma”	4.18 (1, 9)	6.00 (2, 10)	.110
“Follicle Center Lymphoma”	4.08 (1, 9)	6.60 (2, 9)	.056
“Marginal Zone Lymphoma”	3.80 (1, 9)	6.17 (1, 10)	.030†
“Diffuse Large B-Cell Lymphoma”	2.91 (1, 8)	5.50 (1, 9)	.002∗
“CBCL” and *Subtypes*	3.74 (1.00, 8.75)	6.07 (1.50, 9.50)	<.001∗
*All Search Terms*	3.74 (1.00, 8.73)	5.86 (2.00, 9.45)	<.001∗

The above table summarizes search page order of appearance per cutaneous lymphoma “search term” or *group*, stratifying among academic and nonacademic results. Analysis results from 2-sided 2-sample *t* tests for equal variances are reported.

*CBCL*, Cutaneous B-cell lymphomas; *CTCL*, cutaneous T-cell lymphomas.

∗ Indicates significance with *P* < .01.

† Indicates significance with *P* < .05.

Among all academic pages, 56 pertained to “cutaneous lymphoma,” 131 pertained to the CTCL terms, and 72 pertained to the CBCL terms (Supplementary Table I, available via Mendeley at https://data.mendeley.com/datasets/2f8rmmfxm5/1). Among all nonacademic pages, 8 pertained to “cutaneous lymphoma,” 46 pertained to the CTCL search terms, and 33 pertained to the CBCL search terms. Most criteria-excluded CTCL search terms were “folliculotropic mycosis fungoides” (4 academic and 7 nonacademic webpages) and “lymphomatoid papulosis” (9 academic and 5 nonacademic webpages); results mostly qualified for exclusion as academic scientific literature. “Lymphomatoid papulosis” was the only study term to yield <100 results via the search engine, revealing relative dearth of accessible education. All other CTCL search terms, mostly reflecting more common subtypes, resulted in greater qualifying results (eg, at least 20 qualifying for inclusion among academic pages). These results highlight rarer subtypes having more excluded/patient-inaccessible search results, potentiating worse patient comprehension. Comparing CTCL to CBCL, the number of qualifying academic webpages for the “cutaneous T-cell lymphoma” search alone is greater than twice the qualifying academic webpages for the “cutaneous B-cell lymphoma” search (48 vs 22).

Academic pages resulted more frequently than nonacademic pages, with an average order of appearance of 3.74 (range, 1.00-8.73; see Table I). Among nonacademic resources, the average order of appearance was 5.86 (range, 2.00-9.45). “Cutaneous lymphoma” had an average academic search page order of appearance of 4.50 (vs 7.50 for nonacademic, *P* = .003∗); CTCL and subtype search terms had an overall average order of appearance of 3.62 for academic vs 5.73 for nonacademic pages (*P* = .010∗); and CBCL and subtype search terms had an average order of appearance of 3.74 for academic vs 6.07 for nonacademic pages (*P* < .001∗).

Among the 259 academic webpage results, only 27 webpages (10.42%) were hosted by dermatology websites (Supplementary Table I). Among the 87 total nonacademic webpage results, only 3 webpages (3.45%) were hosted by dermatology websites. Instead, most webpages were hosted by sources highlighting an affiliation with oncology (Supplementary Attachment, available via Mendeley at https://data.mendeley.com/datasets/2f8rmmfxm5/1).

### Readability by established grade level and webpage text complexity

Academic vs nonacademic webpage stratification per search term was conducted for separate readability analysis, using established grade level readability calculators (Flesch–Kincaid Grade Level, Gunning Fog Score, SMOG Index, Coleman Liau Index, and Automated Readability Index). Differences among average grade level readability between academic and nonacademic webpages were recorded for “cutaneous lymphoma,” CTCL and subtypes, and CBCL and subtypes (Tables II and III). Among all search terms, average readability grade level was higher for academic result webpages, as compared with nonacademic result webpages, for 3 out of 5 calculators (Flesch–Kincaid, Coleman Liau, and Automated Readability Index); academic webpage average grade levels ranged from 7.92 via Automated Readability Index to 15.79 via Coleman Liau (Supplementary Tables II and III, available via Mendeley at https://data.mendeley.com/datasets/2f8rmmfxm5/1). The other 2 grade level calculators showed nonacademic result webpages as scoring higher in average readability grade levels, 7.84 via SMOG Index and 10.03 via Gunning Fog (Supplementary Tables II and III), as compared with academic result webpages. Average readability of all search terms was found to significantly exceed the AMA’s maximum recommendation of a sixth grade education (*P* < .001∗).

**Table II:** Grade level calculator readability of academic pages

“Search term” or *Group*	Grade level calculators Average (minimum, maximum)				
	Flesch–Kincaid grade level	Gunning fog score	SMOG index	Coleman Liau index	Automated readability index
“Cutaneous Lymphoma”	9.11 (5.10, 15.80)	9.52 (5.30, 17.60)	7.85 (5.10, 13.90)	15.68 (11.60, 23.80)	7.99 (4.00, 16.40)
“CTCL” and *Subtypes*	9.48 (6.88, 16.52)	9.83 (6.00, 16.42)	7.71 (5.72, 12.08)	16.13 (12.72, 24.32)	8.22 (5.15, 15.00)
“CBCL” and *Subtypes*	8.48 (5.63, 13.38)	9.44 (5.63, 15.70)	7.46 (5.63, 11.60)	15.30 (11.70, 20.40)	7.46 (4.85, 12.93)
*All Search Terms*	9.09 (6.26, 15.31)	9.66 (5.80, 16.26)	7.63 (5.63, 12.07)	15.79 (12.25, 22.85)	7.92 (4.94, 14.37)
*All Search Terms* vs AMA recommendation *P* value	<.001∗	<.001∗	<.001∗	<.001∗	<.001∗

The above table summarizes grade level readability of academic result pages for the chosen “search term” or *group* of cutaneous lymphoma terms. Analysis results from 1-sided 1-sample *t* tests against the AMA maximum sixth grade reading level recommendation are reported.

*CBCL*, Cutaneous B-cell lymphomas; *CTCL*, cutaneous T-cell lymphomas.

∗ Indicates significance with *P* < .01.

**Table III:** Grade level calculator readability of non-academic pages

“Search term” or *Group*	Grade level calculators Average (minimum, maximum)				
	Flesch–Kincaid grade level	Gunning fog score	SMOG index	Coleman Liau index	Automated readability index
“Cutaneous Lymphoma”	9.15 (6.70, 12.10)	10.95 (8.40, 14.20)	8.03 (6.40, 10.40)	15.15 (13.20, 18.50)	7.68 (5.90, 10.10)
“CTCL” and *Subtypes*	9.07 (5.83, 13.00)	9.98 (5.77, 14.23)	7.99 (5.68, 11.00)	14.83 (12.40, 18.48)	7.69 (4.17, 11.83)
“CBCL” and *Subtypes*	8.53 (5.18, 12.98)	9.87 (6.10, 14.78)	7.57 (5.48, 10.20)	14.87 (12.10, 18.70)	6.81 (3.70, 11.03)
*All Search Terms*	8.88 (5.67, 12.91)	10.03 (6.13, 14.43)	7.84 (5.67, 10.65)	14.87 (12.36, 18.56)	7.37 (4.15, 11.38)
*All Search Terms* vs AMA recommendation *P* value	<.001∗	<.001∗	<.001∗	<.001∗	<.001∗

The above table summarizes grade level readability of nonacademic result pages for the chosen “search term” or *group* of cutaneous lymphoma terms. Analysis results from 1-sided 1-sample *t* tests against the AMA maximum sixth grade reading level recommendation are reported.

*CBCL*, Cutaneous B-cell lymphomas; *CTCL*, cutaneous T-cell lymphomas.

∗ Indicates significance with *P* < .01.

Academic and nonacademic webpage results were also compared in grade level readability for CTCL search terms vs CBCL search terms. All grade level calculators, besides the Coleman Liau Index, scored CTCL search terms with higher average readability grade level than CBCL search terms; this trend showed higher average readability grade level for CTCL and CBCL search terms’ resulting academic webpages, as compared with respective resulting nonacademic webpages. Among these academic results, average readability grade level for CTCL webpages ranged from 7.71 via SMOG Index to 9.83 via Gunning Fog Index, and for CBCL webpages ranging from 7.46 via SMOG Index to 9.44 via Gunning Fog Index (Supplementary Tables II and III). The Coleman Liau Index for academic webpages for CTCL and subtypes vs CBCL and subtypes showed similar average grade level (16.13 vs 15.30, respectively); for nonacademic webpages, the Coleman Liau grade level index was essentially the same (14.87 vs 14.83, respectively).

Certain cutaneous lymphoma subtypes yielded the highest average readability grade levels. “Lymphomatoid papulosis” scored the highest average readability grade level, ranging from 10.62 (SMOG Index for nonacademic) to 13.50 (Gunning Fog Index for nonacademic), among all grade calculators besides the Coleman Liau Index (Supplementary Tables II and III). The highest average Coleman Liau Index grade was 18.18 for “folliculotropic mycosis fungoides,” as calculated among academic webpages; “Folliculotropic mycosis fungoides” was also among the highest average readability grade levels for the Flesh–Kincaid calculator, with an academic webpage average of 10.20. Interestingly, the Coleman Liau Index calculator scored all search terms (among all webpages) with grade level averages requiring at least 13 years of education, necessitating patients have post-high school education for comprehension.

In addition, webpage text complexity was assessed through crude calculation for each search term, calculating percentage of complex words present on each webpage (Supplementary Tables II and III). CTCL search terms had a higher percentage than CBCL search terms for academic webpages (22.58% vs 20.50%, respectively). However, for nonacademic webpages, CBCL search terms had a slightly higher percentage than CTCL search terms (22.84% vs 21.19%, respectively). “CBCL” had an average text complexity of 26.11% among nonacademic pages and “folliculotropic mycosis fungoides” had an average text complexity of 26.07% among academic pages, representing 2 of the highest percentages of text complexity.

### Overall readability by Flesch–Kincaid Reading Ease

**Fig 1:**
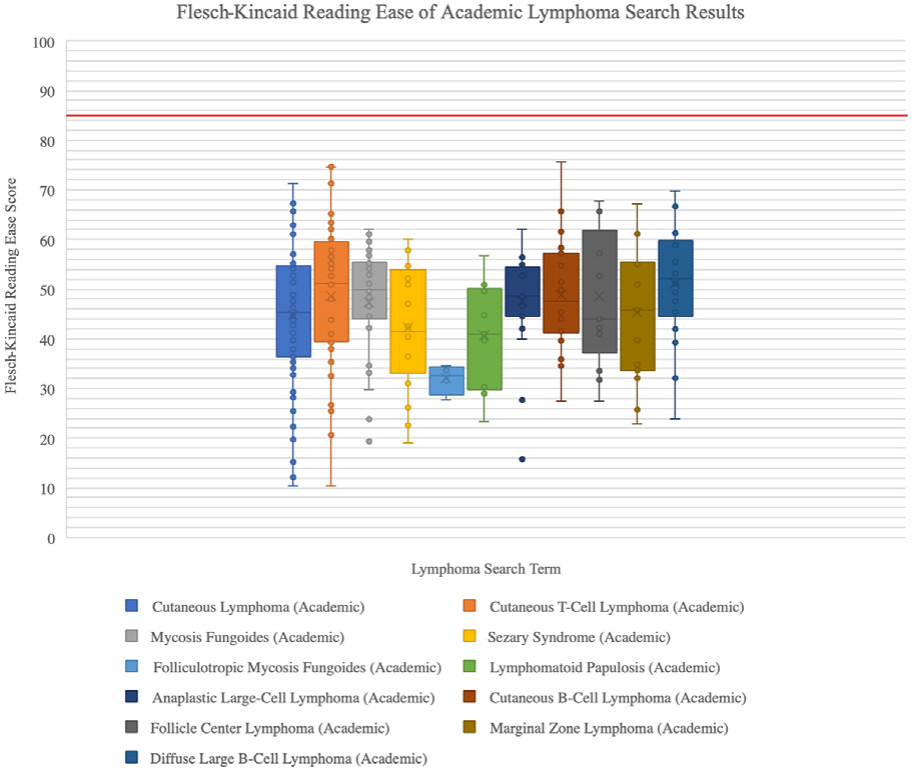
Cutaneous lymphoma search term reading ease of academic pages. The boxplots showcase overall Flesch–Kincaid Reading Ease scores for academic page results of the cutaneous lymphoma search terms. The ease score is based on a ranking of 0 to 100, with lower scores indicating text is more difficult to comprehend and higher scores indicating greater ease of comprehension. The red line indicates a score of 85, between the recommended score range of 80 to 90.

**Fig 2:**
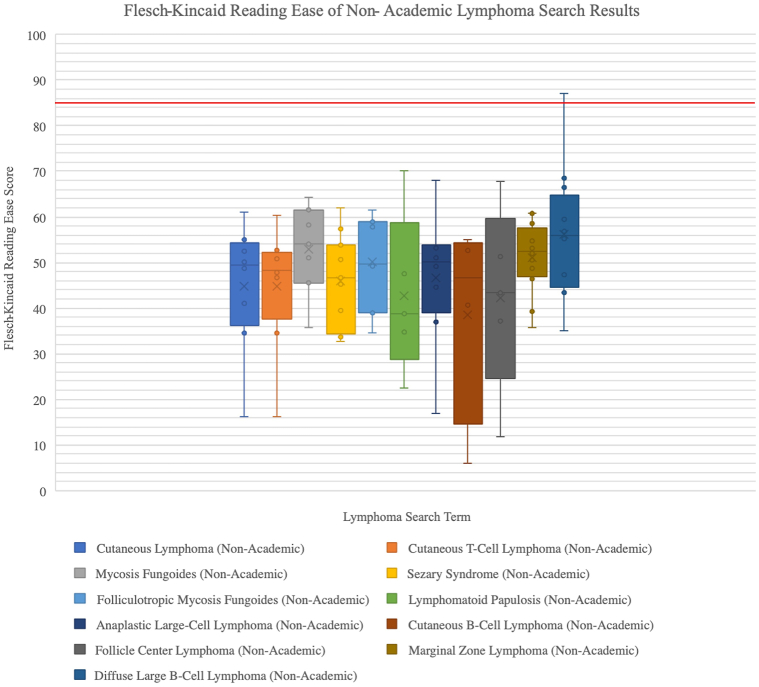
Cutaneous lymphoma search term reading ease of nonacademic pages. The boxplots showcase overall Flesch–Kincaid Reading Ease scores for nonacademic page results of the cutaneous lymphoma search terms. The ease score is based on a ranking of 0 to 100, with lower scores indicating text is more difficult to comprehend and higher scores indicating greater ease of comprehension. The red line indicates a score of 85, between the recommended score range of 80 to 90. Greater variability can be observed among the nonacademic webpages’ box plots, as compared with those of the academic webpages in Figure 1.

Flesch–Kincaid Reading Ease is one of the most widely used measures of readability, even incorporated by United States military to evaluate their manuals.^7^ It is based on ranking between 0 and 100, with lower scores indicating less ease of text comprehension. Among all result pages, medians tended to be approximately 40 to 50, with an average median value of 45.42 for academic pages and 48.65 for nonacademic pages (Figs 1 and 2); this indicates all medians scored within college-level readability (30.0-50.0).^9^ On average, CTCL search terms produced lower median Flesch–Kincaid Reading Ease scores among academic pages, as compared with CBCL search terms (CTCL average of medians 44.11 vs CBCL average of medians 47.41); this trend was also reflected among nonacademic result pages (CTCL average of medians 47.90 vs CBCL average of medians 49.60, see Supplementary Material, available via Mendeley at https://data.mendeley.com/datasets/2f8rmmfxm5/1). Overall, scores should be within 80 to 90 to meet the AMA’s maximum recommendation of a sixth grade education, which all box plots failed to display.^9^

## Discussion

In this study, the accessibility and readability of patient-oriented online education on cutaneous lymphomas were investigated for both CTCL and CBCL subtypes. Search results for the 11 cutaneous lymphoma term searches required extensive exclusion because of abundance of patient-inaccessible scientific literature (Supplementary Attachment). Furthermore, our results suggest that patients with rare subtypes of cutaneous lymphomas not only have fewer patient-oriented educational materials, but less comprehensible materials as well.^2^ Specifically, lymphomatoid papulosis, folliculotropic mycosis fungoides, and anaplastic large-cell lymphoma webpages had the highest average grade level readability of all cutaneous lymphoma subtypes. Future efforts focused on improving resource readability should prioritize these rare subtypes.

Results show earlier order of appearance for academic webpages than nonacademic host webpages. The result is within expectation: search engine prioritizing academic webpages as “most relevant” and/or “most accessed.” Importantly, nonacademic webpages can highlight the patient experience/priorities, warranting study inclusion for accessibility. Our study highlights a dearth of search results from dermatology host websites, with most websites possessing oncology affiliations. One explanation may be that websites and/or organizations dedicated to cutaneous lymphomas are considered more relevant to oncology rather than dermatology (Supplementary Attachment). This could alternatively be attributed to limited representation, research, or sponsorship of cutaneous lymphomas in the field of dermatology; this presents an opportunity for the field to increase such representation—especially since most cutaneous lymphoma care coordination is conducted by dermatologists.

Among readability calculators, cutaneous lymphoma search terms universally produced readability results that significantly exceeded the AMA’s maximum sixth grade recommendation. This study’s results are consistent with published readability literature for pyoderma gangrenosum, urticaria, and even melanoma.^10^, ^11^, ^12^ These studies also found readability of respective online health resources exceeding AMA recommendations. Our study analyzed cutaneous lymphoma resource readability with greater granularity; comparing CTCL vs CBCL and between subtypes, we found CTCL subtype webpages mostly written for slightly higher education levels than for CBCL subtypes. The Flesch–Kincaid Reading Ease and Coleman Liau Index demonstrated readability scores even exceeding high school education; these scores could impress different formulaic approaches, although in line with other scores exceeding AMA recommendations. This trend was consistent across all grade level analytic tools, along with webpages presenting high text complexity.

To rectify this problem, organizations could implement readability evaluations. Websites could earn “certification” if content is presented at an appropriate reading level. Certified websites could be distributed to patients, which would incentivize organizations to improve readability to earn greater patient visibility. To improve readability, we suggest authors identify complex words and replace them with more readable words, possibly using artificial intelligence algorithms as guidance.^13^ For example, the algorithm may switch out “lymphocyte” for “white blood cell” or “malignancy” for “cancer.”

One study limitation is that webpage quality was not evaluated. A recent study focusing on solely the most visible webpages for CTCL found approximately 25% of investigated webpages met Health On the Net code of conduct certification (HONcode), possessing higher quality information and adhering to specified ethical principles. The study found varying quality and popularity of websites, noting highly accessed websites tended to provide reliable patient information.^14^ Accuracy of patient-oriented education may be assessed in future investigations. Additionally, the geolocation of a Google search impacts results.^15^, ^16^, ^17^ Accessibility challenges are amplified for patients that do not speak their country’s native language(s). Moreover, states and countries have different literacy levels, underscoring the equitable issue of readability. Future studies should investigate accessibility and readability from different geolocations or languages.

Given the rarity of cutaneous lymphomas and increasing dependence on online resources for patient education, we urge an increase in patient-oriented education, greater webpage hosting by dermatology host websites, and enhanced readability of existing online patient-oriented education. Overall, this serves as an inflection point for the future of patient education in dermatology: toward pursuit of creating and populating more patient-accessible online education.

## Conflicts of interest

Dr Geskin has served as an investigator for and/or received research support from 10.13039/100008129Helsinn Group, J&J, Mallinckrodt, Kyowa Kirin, Soligenix, Innate, 10.13039/100004334Merck, BMS, and Stratpharma; on the speakers’ bureau for 10.13039/100008129Helsinn Group and J&J; and on the scientific advisory board for 10.13039/100008129Helsinn Group, J&J, Mallinckrodt, 10.13039/100004339Sanofi, 10.13039/100009857Regeneron, and Kyowa Kirin. Authors Schreidah, Fahmy, Lapolla, Gordon, and Dr Kwinta have no conflicts of interest to declare.
